# Molecular characterization of *Babesia microti* thioredoxin (BmTrx2) and its expression patterns induced by antiprotozoal drugs

**DOI:** 10.1186/s13071-018-2619-9

**Published:** 2018-01-15

**Authors:** Jingwei Huang, Kang Xiong, Houshuang Zhang, Yanzhen Zhao, Jie Cao, Haiyan Gong, Yongzhi Zhou, Jinlin Zhou

**Affiliations:** Key Laboratory of Animal Parasitology of Ministry of Agriculture, Shanghai Veterinary Research Institute, Chinese Academy of Agricultural Sciences, Shanghai, 200241 China

**Keywords:** *Babesia microti*, Thioredoxin 2, Antioxidant, Drug response

## Abstract

**Background:**

Human babesiosis is an infectious disease that is epidemic in various regions all over the world. The predominant causative pathogen of this disease is the intra-erythrocytic parasite *Babesia microti*. The thioredoxin system is one of the major weapons that is used in the resistance to the reactive oxygen species (ROS) and reactive nitrogen species (RNS) produced by host immune system. In other intra-erythrocytic apicomplexans like the malaria parasite *Plasmodium falciparum*, anti-oxidative proteins are promising targets for the development of anti-parasitic drugs. However, to date, the sequences and biological properties of thioredoxins and thioredoxin-like molecules of *B. microti* remain unknown. Understanding the molecular characterization and function of *B. microti* thioredoxins may help to develop anti-*Babesia* drugs and controlling babesiosis.

**Methods:**

The full-length *B. microti thioredoxin 2* (*BmTrx2*) gene was obtained using a rapid amplification of cDNA ends (RACE) method, and the deduced BmTrx2 amino acid sequence was analyzed using regular bioinformatics tools. Recombinant BmTrx2 protein was expressed in vitro and purified using His-tag protein affinity chromatography resins. Reverse transcription PCR, quantitative real-time PCR and Western blot were employed to detect the expression and native proteins of BmTrx2. Indirect immunofluorescence assay was used to localize BmTrx2 in *B. microti*. Bovine insulin reduction assays were used to determine the enzyme activity of the purified recombinant BmTrx2 protein.

**Results:**

The full-length *BmTrx2* was 564 bp with a 408 bp open reading frame encoding a protein of 135 amino acids. The predicted molecular weight of the protein was 15.5 kDa. A conserved thioredoxin-like family domain was found in *BmTrx2*. The expression of *BmTrx2* was upregulated on both the third and eighth day post-infection in mice, whereas expression was downregulated during the beginning and later stages. The results of Western blot analysis showed the native BmTrx2 in parasite lysates could be detected by mouse anti-BmTrx2 serum and that the recombinant BmTrx2 protein could be recognized by serum of *B. microti*-infected mice. Immunofluorescence microscopy showed that BmTrx2 localized in the cell cytoplasm of *B. microti* merozoites in *B. microti-*infected red blood cells. The results of bovine insulin reduction assay indicated the purified recombinant BmTrx2 protein possesses antioxidant enzyme activity. Dihydroartemisinin and quinine, known anti-malaria drugs, and clindamycin, a known anti-babesiosis drug, induced significantly higher upregulation of *BmTrx2* mRNA.

**Conclusions:**

Our results indicate that *BmTrx2* is a functional enzyme with antioxidant activity and may be involved in the response of *B. microti* to anti-parasite drugs.

**Electronic supplementary material:**

The online version of this article (10.1186/s13071-018-2619-9) contains supplementary material, which is available to authorized users.

## Background

*Babesia microti* is a tick-borne protozoan parasite belonging to the phylum Apicomplexa that invades and replicates in the erythrocytes of animals, including humans, causing zoonotic babesiosis [1]. There are over 100 different species of the genus *Babesia*, including *B. microti*, *B. bovis*, *B. divergens* and *B. bigemina*, that range in size between 1 and 5 μm [2]. Since the first report of human infection with *Babesia* in 1957, thousands of human infection cases have been reported around the world, making babesiosis an important emerging human infectious disease [3]. The most common symptoms of severe infection *Babesia* are hemolytic anaemia and jaundice. Severe symptoms occur in susceptible patients, such as those who are asplenic or immunosuppressed. In 2011, the first two humans infected with *B. divergens* in China were reported in Shandong Province [4]. Given its prevalence and severity, and because there are currently no effective drugs to treat babesiosis, the need for the identification of new drug targets is urgent.

Reactive oxygen species (ROS) and reactive nitrogen species (RNS) in the high oxygen environment of mammalian hosts’ red blood cells (RBCs) are toxic to *Babesia* trophozoites and merozoites. Therefore, to survive in the host, the parasites must protect themselves from elimination by ROS [5]. However, because these parasites lack antioxidant enzymes, such as catalase and glutathione peroxidase, the thioredoxin system plays a significant role in their resistance to host oxidative stress [6, 7]. The thioredoxin system consists of three major components, thioredoxin (Trx), thioredoxin reductase (TrxR) and NADPH, of which Trx plays a central role in redox regulation and antioxidation [8]. Other apicomplexan parasites, such as *Plasmodium falciparum*, are sensitive to disturbances in the antioxidant system, making it a potential target for novel antiprotozoal drugs [7]. Previous studies showed that five Trx genes had been found in *P. falciparum*. Among them, thioredoxin 1–3 can be reduced by PfTrxR; however thioredoxin-like protein 1 and 2 cannot [9]. Trx has already been studied as a potential drug target [7]. In *Babesia*, other antioxidant molecules have been reported, including superoxide dismutase, catalase, and glutathione reductase [10, 11]. However, no research on the thioredoxins of *Babesia* has been reported. In the present study, the *B. microti Trx2* (*BmTrx2*) gene was cloned and characterized, and its role in response to antiprotozoal drugs was tested*.*

## Methods

### Parasites and animals

The *B. microti* strain ATCC® PRA-99TM was obtained from the American Type Culture Collection (Manassas, VA, USA) and maintained in our laboratory by serial passage in BALB/C mice (SLAC, Shanghai, China) using the method described previously [12].

### Transcriptome sequencing of *B. microti*

The *B. microti* total RNA sequencing using Illumina Hiseq 4000 was performed by BGI (The Beijing Genomics Institute, Shenzhen, China) as described previously [12].

### Molecular cloning and sequence analysis of *BmTrx2*

*BmTrx2* was selected from the cDNA library constructed previously, for further study. Total RNA was extracted from erythrocytic stage *B. microti* using a routine method as described elsewhere [12]. For the synthesis of complementary DNAs (cDNAs), reverse transcription PCR (RT-PCR) was performed using PrimeScript™ RT reagent Kit with gDNA Eraser (TaKaRa, Dalian, China), according to the instructions. The partial coding sequence of *BmTrx2* has been deposited in the GenBank database (reference sequence: XM_021482575.1) [13]. However, the complete open reading frame (ORF) of *BmTrx2* was not available. The full-length *BmTrx2* was generated by rapid amplification of cDNA ends (RACE) using SMARTer RACE cDNA amplification kit (Clontech, Mountain view, USA) according to the manufacturer’s instructions. The full-length *BmTrx2* ORF was amplified using primers designed based on the sequence which was generated by RACE. The primer sets used in this study are shown in Table 1. The amplified PCR products were subsequently purified and subcloned into the pMD-19 T vector (TaKaRa) and further confirmed by sequencing, which was performed at Genewiz (Genewiz Inc., Suzhou, China). Finally, the identified sequence was submitted to the NCBI database.

**Table 1:** Oligonucleotide primer sequences used for PCR in this study

Name	Sequence (5'-3')	Description
BmTrx2-Con-F	GCACTACACATACCCACGTATTAT	Forward primer specific for BmTrx2 conserved sequence (partial)
BmTrx2-Con-R	TGCGTCAATTCCAGTGACAATTAC	Reverse primer specific for BmTrx2 conserved sequence (partial)
3' GSP1	CATAGAGACAGTGGATTGCTACAGG	Forward gene specific primer for 3'-end of BmTrx2 in primary PCR
3' GSP2	GAGCACAGAGTTACTACGATTCCCAT	Forward gene specific primer for 3'-end of BmTrx2 in second PCR
5' GSP1	CCGATTCCAACTTAGG	Gene specific primer for reverse transcription of *B. microti* mRNA
5' GSP2	GGGAATCGTAGTAACTCTGTGCTCC	Reverse gene specific primer for 5'-end of BmTrx2 in primary PCR
5' GSP3	GCCTTCCTGTAGCAATCCACTGTC	Reverse gene specific primer for 5'-end of BmTrx2 in second PCR
BmTrx2-ORF-F	CATATGCATAGCATGAGTAGGGTCATATTT	Forward primer containing an *Nde*I site for cloning into pET30a(+)-BmTrx2
BmTrx2-ORF-R	CTCGAGCTTTTGAGGGGGTGTGACGTGT	Reverse primer containing a *Xho*I site for cloning into pET30a(+)-BmTrx2
BmTrx2-qRT-F	GTCTAGTGGCGTTGTTGTTGC	Forward gene specific primer for the quantification of BmTrx2 mRNA
BmTrx2-qRT-R	GACCGATTTCATCTGATTGCTTA	Reverse gene specific primer for the quantification of BmTrx2 mRNA
Bm18S-qRT-F	GTTATAGTTTATTTGATGTTCGTTT	Forward gene specific primer for the quantification of *B. microti 18S* mRNA
Bm18S-qRT-R	AAGCCATGCGATTCGCTAAT	Reverse gene specific primer for the quantification of *B. microti 18S* mRNA

The software Genetyx (Software Development Co., Ltd., Tokyo, Japan) was used to analyze the nucleotide and the deduced amino acid sequences. As for sequence homology, Basic Local Alignment Search Tool (BLAST; http://www.ncbi.nlm.nih.gov/blast/Blast.cgi) was employed for the assessment. The phylogenetic tree was constructed using ClustalW alignment and neighbour-joining method of the software Mega 6.06.

### In vitro expression and purification of recombinant BmTrx2 protein

The ORF of *BmTrx2* was amplified by polymerase chain reaction (PCR) using the previously described primers and directionally cloned into the pET-30a(+) vector (Novagen, Madison, USA). The recombinant plasmid pET-30a(+)-BmTrx2 was verified by sequencing and then transferred into *Escherichia coli* BL21 (DE3) (Novagen) for expression of a six His-tagged recombinant protein. Positive clones were cultured in LB media and induced with the isopropyl β-D-thiogalactoside (IPTG) (1μg/ml) at 16 °C for 12 h (optical density at 600 nm OD_600_ = 0.6).

Recombinant BmTrx2 protein was purified using Ni-NTA His·Bind Resin (Millipore, Burlington, USA), following instructions of the manufacturer. The purity of the purified protein was confirmed by SDS-PAGE on 12% electrophoretic gels, and the concentration was measured using a Pierce BCA Protein Assay Kit (Thermo Fisher Scientific, Waltham, USA). Pierce High Capacity Endotoxin Removal Spin Columns (Thermo Fisher Scientific) was utilized to remove the endotoxin from protein samples used for in vivo trials. The purified protein was aliquoted and stored at -80 °C for further use.

### Immune serum production

A polyclonal antibody against rBmTrx2 was obtained using a method described previously [14] except the purified rBmTrx2 were used in our study. The serum was collected 2 weeks after the third immunization. Specific anti-*BmTrx2* antibody titers were assessed by enzyme-linked immunosorbent assay (ELISA) [15].

Anti-*B. microti* serum was collected from BALB/C mice 21 days post-infection with 1 × 10^8^ *B. microti*-infected RBCs (iRBCs) with a parasitemia of 30%. The immune serum was aliquoted and stored at -80 °C until further use.

### Western blot analysis of the native and recombinant BmTrx2 proteins

The Western blot analysis was performed using a regular method described previously [14]. Briefly, samples from five batches of somatic extract of iRBCs (5, 6, 7 and 8 days post-infection and non-infected erythrocytes as a negative control) and the purified rBmTrx2 were fractioned by SDS-PAGE. Gel-separated proteins were then transferred to polyvinylidene fluoride (PVDF) membranes (Millipore). Then, primary antibodies, mouse anti-rBmTrx2 sera (dilutions 1:200) or mouse anti-*B. microti* sera (dilutions 1:200) were applied, respectively. Finally, a secondary antibody HRP-conjugated goat-anti-mouse IgG (dilutions 1:2000) (Sigma-Aldrich, St. Louis, USA) was added and the protein-antibody complex was developed using an Enhanced DAB kit (Tiangen Biotech, Beijing, China) following to the manufacturer’s instructions.

### Relative expression analysis of *BmTrx2* post-infection

The determination of BmTrx3 expression post-infection was performed as described previously [16] except the *18S* ribosomal RNA of *B. microti* (*Bm18S*) (GenBank: XM_021481625.1) [13, 17] was used as an internal control in this study. Briefly, Mice were injected with 1 × 10^8^ iRBCs, and the blood was collected from 1 to 10 days post-injection. Total RNAs were extracted from iRBCs at different post-infection time points for qRT-PCR analysis. The specific primers used to quantify *BmTrx2* and *Bm18S* are shown in Table 1. Also, the parasitemia was calculated at each day post-infection.

### Expression of *BmTrx2* after treatment with antiprotozoal drugs

In this study, a *B. microti* iRBCs short-term in vitro culture system was established. Briefly, *B. microti-*infected mice blood (30% parasitemia) was collected by cardiac puncture after anaesthesia. iRBCs were washed four times with sterile PBS. For drug screening, a 12-well flat-bottom plate (Nunc cell culture, USA) was used. A total of 2 × 10^7^ *B. microti* iRBCs were cultured in basic media (RPMI 1640 containing 40% fetal bovine serum [FBS], 25 mM HEPES) at 37 °C in an atmosphere of 95% air and 5% CO_2_ [18, 19].

To assess the effect of drugs on *BmTrx2* gene expression, the short-term in vitro growth of iRBCs against four antiprotozoal drugs (dihydroartemisinin, quinine, chloroquine and clindamycin) (Sigma-Aldrich) was tested. Dimethylsulfoxide (DMSO) was used to dissolve dihydroartemisinin, quinine, and clindamycin, whereas PBS was utilized to dilute chloroquine. iRBCs were treated with different concentrations (20, 50, or 100 μM) of dihydroartemisinin, quinine, chloroquine, and clindamycin for 24 h, and controls were treated with DMSO or PBS. iRBCs were processed as described above and relative *BmTrx2* transcript levels were assessed.

### Indirect immunofluorescence assay

The indirect immunofluorescence assay was conducted using a conventional method described elsewhere [16] with slight modifications. Serum of mice hyper-immunized against rBmTrx2 was generated by immunizing animals three times. *B. microti* iRBCs smears were fixed with methanol and acetone (1:1, *v*/v) and permeabilized with 0.1% TritonX-100. After washing, primary antibody, mouse anti-BmTrx2 serum diluted 1:500 in 3% BSA was applied. The secondary antibody used in this study was Alexa-Fluor®488 conjugated goat anti-mouse IgG (Invitrogen, Carlsbad, USA). Nucleus stain was performed by incubating the smears with 2 μg/ml Hoechst 33342 (Thermo Fisher). Finally, the slides were examined using a confocal laser-scanning microscope (Zeiss LSM 880, Oberkochen, Germany) after mounting with Fluoromount Aqueous Mounting Media (Sigma-Aldrich). The parameters in all the experiment and control groups were set at the same values.

### Enzyme activity assays

The evaluation of rBmTrx2 enzyme activity was conducted using a bovine insulin reduction assay described previously [20]. Briefly, 16 mM bovine insulin (Sigma-Aldrich), 10 μM purified rBmTrx2, 100 nM rat thioredoxin reductase (rat TrxR), 100 μM NADPH and 2 mM EDTA in 50 mM Tris-HCI (pH 6.5) were mixed in a reaction system. Thioredoxin activity was determined by measuring the decrease in absorbance at 340 nm. His fusion protein (10 μM) and His-tagged *E. coli* thioredoxin (10 μM) were set as controls. All reactions were performed in triplicate for each enzyme.

### Data analysis

A GraphPad PRISM 5 software (GraphPad Software Inc., CA, USA) was employed for the statistical analysis. The mean ± standard error (SEM) of each group was calculated. The differences between groups were determined using two-tailed t-tests. *P* < 0.05 was considered significant and *P* < 0.01 was considered highly significant.

## Results

### Cloning of the *BmTrx2* gene

Transcriptome sequencing of *B. microti* revealed 3024 genes obtained from the transcriptome assembly. The partial coding sequences of a thioredoxin reductase and six thioredoxins were present. By multiple alignments using BLAST, these identified genes were named as *B. microti thioredoxin reductase 1* [20] and *Trx 1–6* (GenBank: KX758048–KX758053) based on the similarity to the thioredoxin genes found in the open database of apicomplexans including *Babesia*, *Plasmodium* and *Theileria*. For the present research, *BmTrx2* (KX758049.1) (https://www.ncbi.nlm.nih.gov/nuccore/1235975689) was chosen for further study.

**Fig. 1:**
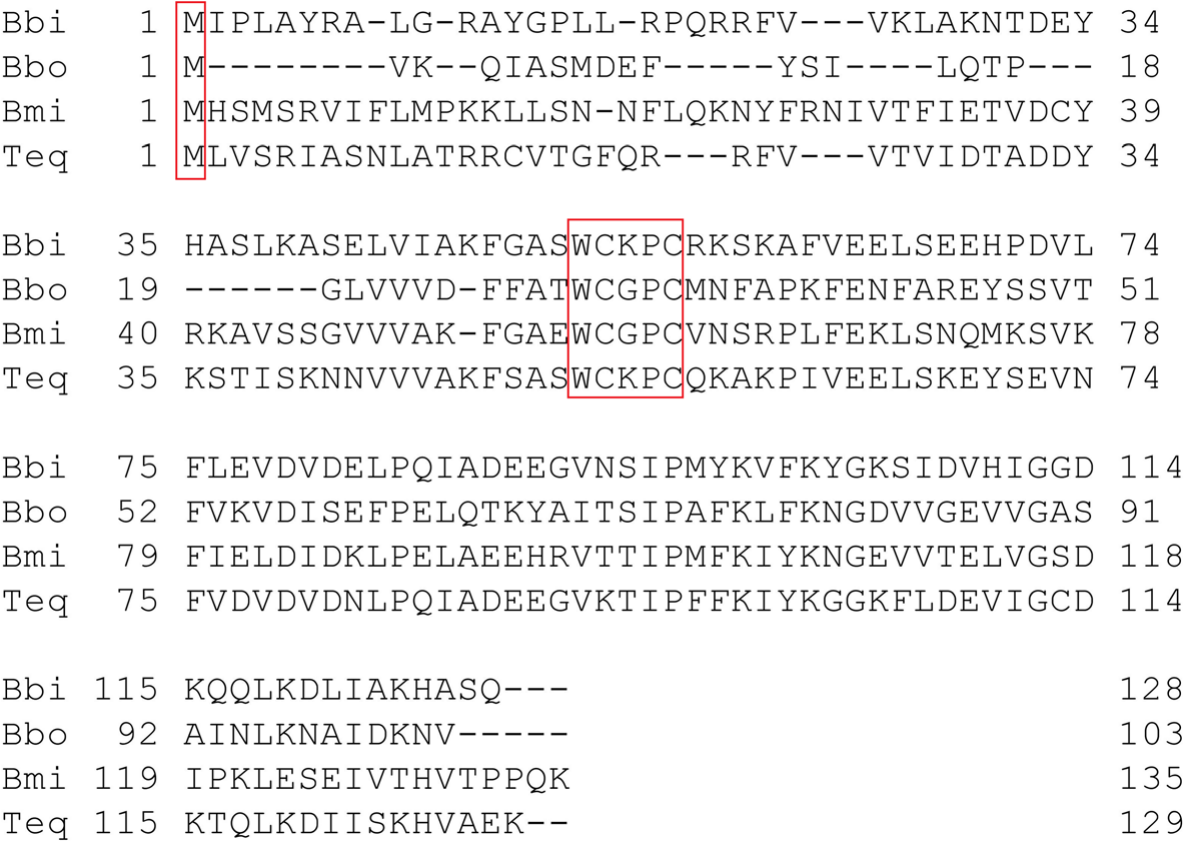
The deduced amino acid sequence of *BmTrx2* and the multiple alignments. The conserved thioredoxin-like family domain was shown in the box. *Abbreviations*: Bbi, *Babesia bigemina* (XP_012765929); Bbo, *Babesia bovis* (XP_001609888); Bmi, *Babesia microti* (KX758049.1); Teq, *Theileria equi* (XP_004828582)

**Fig. 2:**
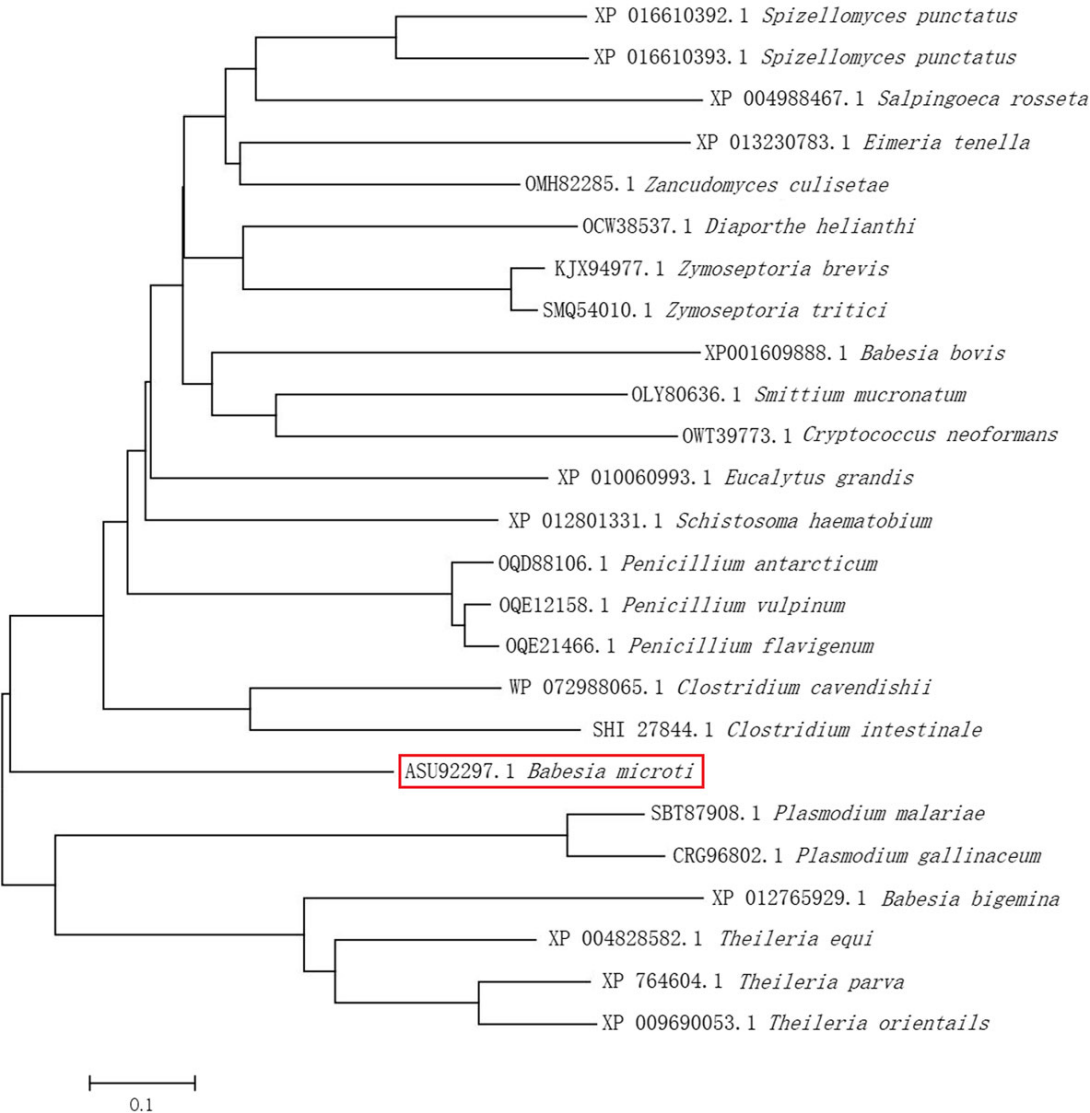
Phylogenetic tree on the basis of alignment of thioredoxin sequences using Mega 6.06

Results of RACE and sequencing showed that the full-length *BmTrx2* gene was 564 bp with an ORF of 408 bp encoding a protein of 135 amino acids. Bioinformatics predicted no signal peptides within the *BmTrx2* protein. The molecular weight of the predicted protein was 15.5 kDa, and the isoelectric point was 8.77. The results of multiple alignments revealed that the amino acids sequence of the *BmTrx2* protein shared 38% similarity with *B. bigemina Trx* (XP_012765929), 45% similarity with *B. bovis Trx* (XP_001609888), and 45% similarity with *Theileria equi Trx* (XP_004828582) (Fig. 1). The results of the phylogenetic analysis revealed that *BmTrx2* was most closely related to *Theileria* (Fig. 2). A thiol-disulfide redox active center (-WCGPC-), which is the typical catalytic domain of Trx, was identified in the amino acid sequence of the BmTrx2 protein (Fig. 1). These results showed that the *Trx2* obtained from the mRNA of *B. microti* merozoites is a typical thioredoxin family protein.

### In vitro expression and purification of rBmTrx2

**Fig. 3:**
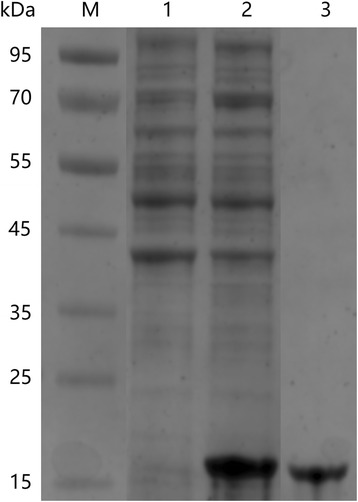
In vitro expression and purification of recombinant *BmTrx2*. Lane M: standard protein molecular weight marker; Lane 1, induced cell lysates; Lane 2: precipitation of induced cell lysates; Lane 3: purified rBmTrx2

The recombinant BmTrx2 protein was successfully expressed in *E. coli* as a His-tagged fusion protein and purified using Ni-affinity chromatography. SDS-PAGE results showed that the molecular weight of rBmTrx2 was 15.5 kDa, which was in accordance with the predicted size (Fig. 3). Purified rBmTrx2 were used to obtain mouse anti-BmTrx2 polyclonal serum after 3 immunizations.

### Serological detection of native and recombinant BmTrx2 protein

**Fig. 4:**
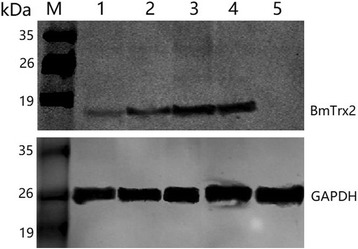
Western blot analysis of the native *BmTrx2*. Lane M: standard protein molecular weight marker; Lanes 1–4: *B. microti* infected mouse erythrocyte lysates on 5th, 6th, 7th and 8th day post-infection; Lane 5: uninfected mouse erythrocyte lysate; mouse anti-rBmTrx2 serum was used as primary antibody in this Western blot analysis

An antiserum obtained from mice immunized with rBmTrx2 was utilized to detect the native *BmTrx2* protein. The results of Western blot assays showed that a specific protein band of approximately 15.5 kDa was detected in the iRBC lysates 5, 6, 7 and 8 days post-infection, whereas no bands were detected in the un-infected homogenates (Fig. 4). The molecular size of the detected bands was in line with the expected size of the natural *BmTrx2.* No other bands were recognized by the antiserum.

**Fig. 5:**
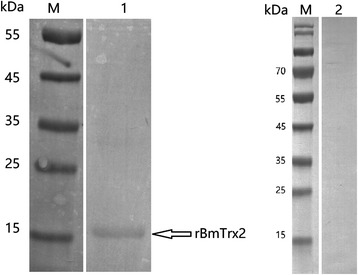
Western blot analysis of the recombinant BmTrx2 protein. Lane M: standard protein molecular weight marker; Lane 1: purified rBmTrx2 probed by serum from mice infected by *B. microti* on 21st d; Lane 2: purified rBmTrx2 probed by uninfected mouse serum; the samples and markers were transferred into the same membrane, after that each lane was separated

Western blot analysis also indicated that rBmTrx2 was identified by serum of mice experimentally infected with *B. microti*, whereas the serum of non-infected mice did not detect rBmTrx2 (Fig. 5). These results suggest that BmTrx2 protein has high immunogenicity and can induce hosts immune response.

### Relative expression analysis of *BmTrx2* post-infection

**Fig. 6:**
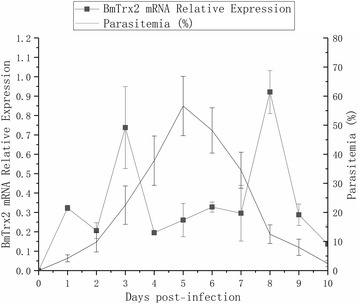
Relative expression analysis of *BmTrx2* on different days post-infection. Expression analysis of *BmTrx2* with parasitemia on different days post-infection

To investigate the expression profile of *BmTrx2* over time, total RNA of iRBCs from different days post-infection was assessed by qRT-PCR analysis. *BmTrx2* expression peaked 3 days post-infection and declined suddenly until 7 days post-infection. Eight days post-infection, the relative expression of *BmTrx2* peaked again and then rapidly decreased to low levels (Fig. 6).

Blood obtained via the caudal vein was used to prepare smears which were stained with Giemsa to assess the advancement of the infection by calculating the ratio of iRBCs. Starting on the first day post-infection, *B. microti* was detected in iRBCs. The parasitemia ratio increased until 5 days post-infection, after which it started to decline until reaching undetectable levels by day 10 post-infection. No parasites were detected in the uninfected mice throughout the selected time points (Fig. 6).

### *BmTrx2* response to chloroquine, clindamycin, dihydroartemisinin and quinine

**Fig. 7:**
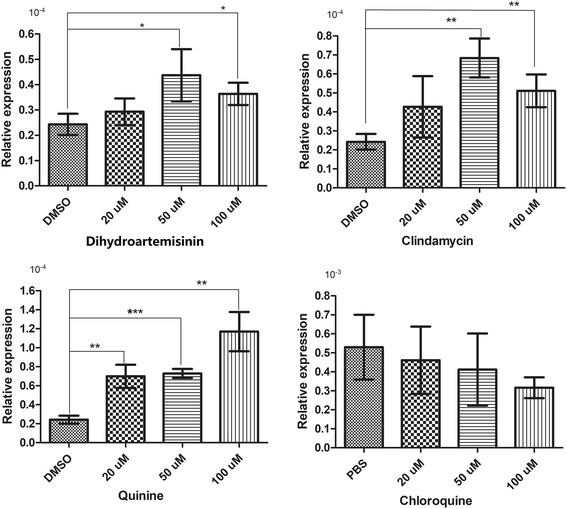
Relative expression analysis of *BmTrx2* in iRBCs exposed to anti-parasitic agents for 24 h. The iRBCs was treated with different concentrations (20 μM, 50 μM, 100 μM) of chloroquine, clindamycin, quinine and dihydroartemisinin, respectively

mRNA relative expression levels of *BmTrx2* were investigated after subjected to different doses of chloroquine, clindamycin, dihydroartemisinin or quinine for 24 h (Fig. 7). Results showed that *BmTrx2* was significantly upregulated after treatment with dihydroartemisinin, clindamycin or quinine compared with the control group, whereas no significant difference was observed after chloroquine treatment (Additional file 1: Table S1). Furthermore, quinine-induced *BmTrx2* expression was dose-dependent (Fig. 7). These findings indicate that *BmTrx2* might be implicated in the response of *B. microti* to clindamycin, dihydroartemisinin and quinine.

### Immunofluorescence assays

**Fig. 8:**
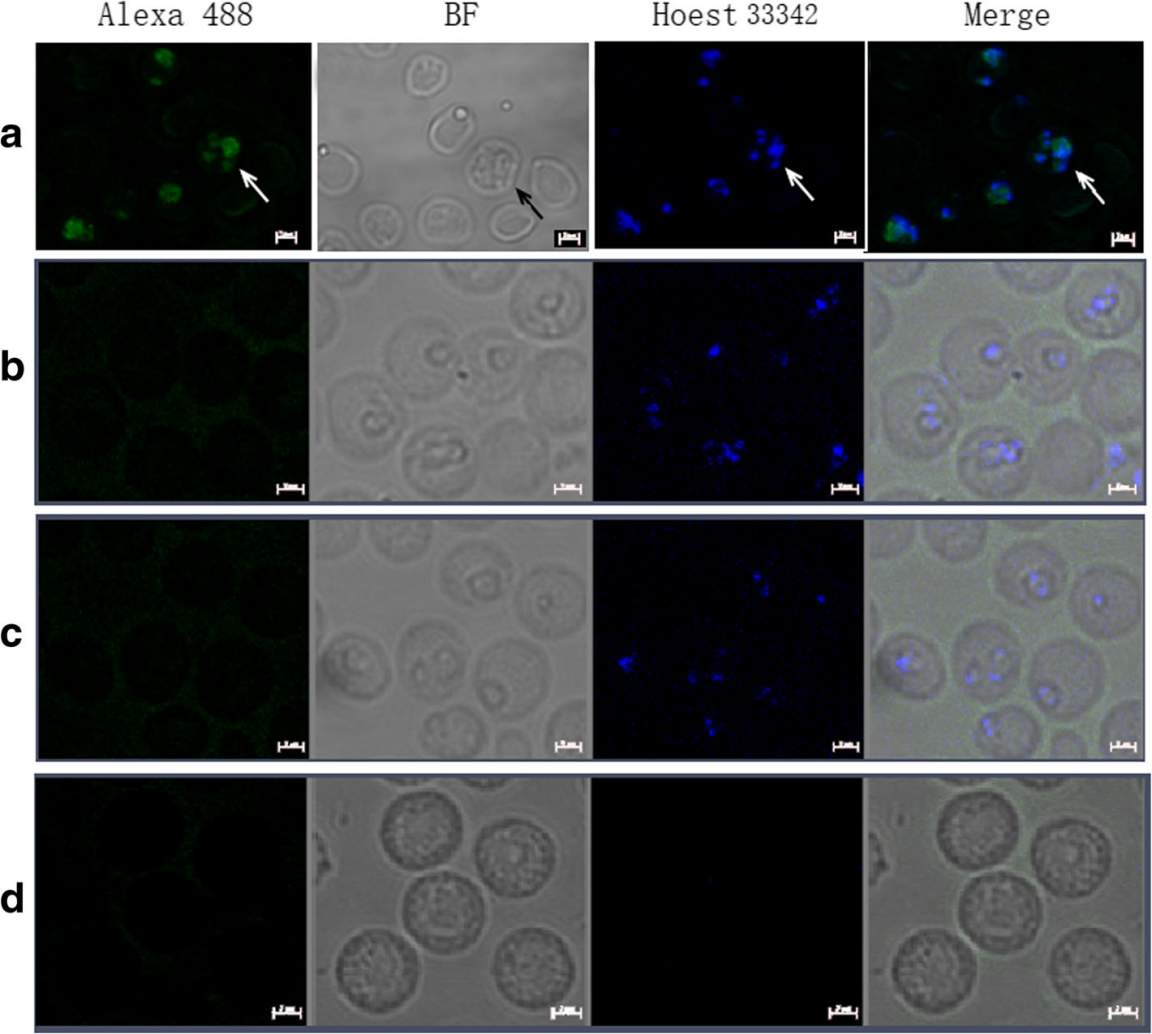
Immunofluorescence microscopy of native *BmTrx2*. Observation of the native *BmTrx2* recognized by mouse anti-rBmTrx2 serum in confocal laser micrographs (**a**) iRBCs incubated with mouse anti-rBmTrx2 serum as primary antibody; (**b**) iRBCs incubated with normal mouse serum as primary antibody; (**c**) iRBCs incubated with only secondary antibody; (**d**) uninfected RBCs incubated with mouse anti-rBmTrx2 serum as primary antibody; *Scale-bars*: 2 μm

The subcellular location of BmTrx2 in *B. microti* was determined by indirect immunofluorescence assays. *B. microti*-infected RBCs (approximately 20% parasitemia) and mouse anti-rBmTrx2 serum were used. As shown in Fig. 8, blue fluorescence represents the nucleus of *B. microti* cells and, green fluorescence represents BmTrx2 located around the nucleus of *B. microti* merozoites in iRBCs (Fig. 8a). In contrast, no green fluorescence was detected in the iRBCs incubated with non-immune mouse serum (Fig. 8b). Similarly, only blue fluorescence was detected in iRBCs incubated with only second antibody (Fig. 8c). No fluorescence staining was visualized in control mice RBCs incubated with mouse anti-rBmTrx2 serum (Fig. 8d). These results indicate that BmTrx2 is expressed in the cytoplasm of *B. microti* merozoites.

### Determination of enzyme activity

**Fig. 9:**
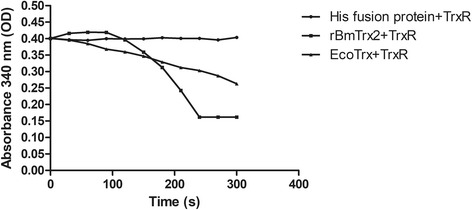
The enzyme activity curve of the recombinant BmTrx2 protein

Enzyme activity of purified rBmTrx2 was analyzed by bovine insulin reduction assays. His-tag was fused to rBmTrx2 protein, and His-tag polypeptide was used as a control. As shown in Fig. 9, absorbance of the His-tag polypeptide remained stable throughout the assay, suggesting that His-tagged polypeptide has no enzymatic activity. In comparison to the positive control (*E. coli* Trx and rat TrxR), the results showed that the activity of rBmTrx2 was low for the first 100 s of the assay. The absorbance at 340 nm of NADPH gradually decreased at 100–240 s indicating that rBmTrx2 did possess enzymatic activity. After 240 s, the absorbance remained at low levels.

## Discussion

*Babesia microti*, similar to the malaria parasite *P. falciparum*, is highly equipped with tools to counter the impact of the oxidative stress when the parasites are exposed during the erythrocytic stages. Because intracellular parasites like these apicomplexan protozoa lack antioxidant enzymes such as catalase and glutathione peroxidase, they rely on the thioredoxin system to resist host oxidative stress [21]. Thioredoxin (Trx), thioredoxin reductase (TrxR) and NADPH are involved in the thioredoxin system, and they are widely present in prokaryotes and eukaryotes. The multifaceted functions played by Trx that indirectly depend on TrxR mainly include the modification of the redox status of protein targets via a Cys-xx-Cys active site motif [8]. In this investigation, an essential component of the *B. microti* thioredoxin system, *BmTrx2* was characterized, and the biochemical properties were identified.

Survival of intraerythrocytic *P. falciparum* emphasized and confirmed how essential thioredoxin reductase is. Previous studies have proved the essential role of thioredoxin reductase in *P. falciparum* and also the crucial significance of the thioredoxin redox cycle [22]. Other studies reported that Trxs had been identified as potential drug targets for the development antimalarial drugs [23]. In *P. falciparum*, three classic thioredoxins (Trx 1–3) and two thioredoxin-like proteins (Tlp1 and Tlp2) have been identified [8]. Similarly, six thioredoxin-associated genes were identified in the transcriptome of the *B. microti*. Using BLAST, the sequence identity of *B. microti Trx2* and *B. bigemina Trx* (XP_012765929) was 38% showing a low homology. However, a typical thiol-disulfide redox active center -Trp-Cys-Gly-Pro-Cys- (-WCGPC-), the Trx catalytic domain critical for regulation of the formation of redox-active disulfide bonds, was identified in the sequence of *BmTrx2*. The highly conserved Trp and Asp residues provided a hydrogen bonding so that the active site is stable [24]. Also, other studies reported that the highly conserved domain is essential in the maintenance of the three-dimensional structure of Trx [25]. In our study, sequence analysis revealed that the redox active center of *BmTrx2* (-WCGPC-) was the same as that of *PfTrx1*, and different from that of *PfTrx2* (-WCQAC-). The results of sequence alignments showed that *BmTrx2* amino acid sequence shared a higher similarity with *PfTrx1* (39%) than with *PfTrx2* (26%). In the present study, we also showed that rBmTrx2 could transfer electrons to bovine insulin and be reduced by TrxR and NADPH. These results indicated that *BmTrx2* might function in the regulation of redox balance and the thioredoxin systems of *B. microti* and *P. falciparum* were different from each other even though they share almost the same life-cycle during the erythrocytic stage.

In our Western blot experiments, a specific band was detected in the lysates of *B. microti*-infected RBCs. The molecular weight of native BmTrx2 was 15.5 kDa which was consistent with the size of the recombinant *B. microti* Trx2 protein. The results of the Western blot experiments showed that the expression of BmTrx2 was gradually upregulated and reached a peak at eight days post-infection. On the other hand, the results of qRT-PCR showed that the expression of BmTrx2 peaked at both three and eight days post-infection, respectively. These results are in accordance with each other. At three days post-infection, *B. microti* was undergoing a rapid propagation process in the host RBCs since the parasitemia increased quickly. Since it has been reported that native *B. microti* Trx protects DNA from oxidative damage and RBCs are oxygen-rich environments, *B. microti* must secrete large amounts of Trx to exert antioxidant effects [26, 27]. At a rational point of view, at three days post-infection, high expression levels of BmTrx2 helped *B. microti* maintain redox balance during the rapid propagation process. The *B. microti* parasitemia at eight days post-infection decreased to a level of 12.5% and subsequently maintained low levels. However, the level of BmTrx2 expression reached a peak at the same time. Interestingly, similar results were also observed in previous studies on other *B. microti* antioxidant enzymes such as peroxiredoxin 2 [14] and aldo-keto reductase-like protein [16]. This might be because of the host immune elimination. However, the molecular mechanisms of the high-level expression of BmTrx2 at eight days post-infection need to be further investigated.

Previous studies reported that *P. falciparum* Trx2 was located in mitochondria [28]. However, more recently, other studies revealed that PfTrx2 is part of an export machinery called the translocon of exported proteins (PTEX), which is located in the parasitophorous vacuole membrane [29, 30]. In the present study, indirect immunofluorescence assays were performed to localize BmTrx2 in *B. microti* merozoites. The fluorescence pattern detected suggested that the target molecules are located in the cytoplasm. However, the precise location of BmTrx2 still needs to be confirmed by co-localization with multiple markers, and molecular function of this gene needs further investigation.

In *P. falciparum*, *PfTrx2* plays a role in the regulation of PTEX-mediated protein export and is proved to be essential in maintaining normal erythrocytic stage growth [31]. As a crucial nexus for protein export in malaria parasites, Trx2 is identified to be a potential drug target [32]. Previous studies revealed that targeting PfTrx2 pharmacologically might interfere with protein secretion, suggesting a new strategy for antimalarial drug development [33]. However apicomplexan parasites employ various mechanisms of drug resistance [34, 35], therefore, to explore the role of *BmTrx2* in response to anti-babesiosis drugs and to enhance the potential of chemotherapy against babesiosis, we measured the relative expression of BmTrx2 when the parasite was subjected to chloroquine, clindamycin, quinine, and dihydroartemisinin. Our results showed that clindamycin, quinine, and dihydroartemisinin could promote BmTrx2 transcription, indicating *BmTrx2* might have a role in the way these pharmaceutical agents act. However, the exact molecular mechanism of how *BmTrx2* function in response to the antiprotozoal drugs still needs further exploration.

## Conclusions

To our knowledge, this is the first report to identify, characterize, and functionally analyze *Trx* from *B. microti*. Our results showed that *BmTrx2* is an antioxidant gene with a conserved thioredoxin domain that could be used as a potential target for the development of new drugs against babesiosis.

## Additional file

Additional file 1: Table S1. Detailed statistical analysis of Fig. 7. This file provides the statistical analysis details of the comparison between groups in the drug response assay. (DOCX 16 kb)

## Abbreviations

*BmTrx2*: *B. microti* thioredoxin 2
DAB: 3, 3′-diaminobenzidine tetrahydrochloride
Hoechst 33,342: 2′-[4-ethoxyphenyl]-5-[4-methyl-1-piperazinyl]-2,5′-bi-1H-benzimidazole trihydrochloride trihydrate
IFA: Immunofluorescence assay
IPTG: Isopropyl β-D-1-Thiogalactopyranoside
LB: Luria-Bertani
PBS: Phosphate-buffered saline
qRT-PCR: Quantitative real-time PCR
RACE: Rapid amplification of cDNA ends
SDS-PAGE: Sodium dodecyl sulfate-polyacrylamide gel electrophoresis
Trx: Thioredoxin
TrxR: Thioredoxin reductase
