# Supplementary material for: Molecular characterization of Babesia microti thioredoxin (BmTrx2) and its expression patterns induced by antiprotozoal drugs
Source: Parasit Vectors. 2018 Jan 15;11:38. doi: 10.1186/s13071-018-2619-9 (PMC5769273; doi:10.1186/s13071-018-2619-9)
Supplement: Additional file 1: Table S1. — Detailed statistical analysis of Fig. 7. This file provides the statistical analysis details of the comparison between groups in the drug response assay. (DOCX 16 kb) [file 13071_2018_2619_MOESM1_ESM.docx]

**Statistical analysis in detail**

**Fig. 7**

**Dihydroartemisinin:**

|  | t, df | P value | P value summary | Are means signif. different? (P < 0.05) | One- or two-tailed P value? |
| --- | --- | --- | --- | --- | --- |
| DMSO vs 20 μM | t=1.279 df=4 | 0.2700 | ns | no | Two-tailed |
| DMSO vs 50 μM | t=3.026 df=4 | 0.0389 | * | yes | Two-tailed |
| DMSO vs 100 μM | t=3.441 df=4 | 0.0263 | * | yes | Two-tailed |
| 20 μM vs 50 μM | t=2.160 df=4 | 0.0969 | ns | no | Two-tailed |
| 20 μM vs 100 μM | t=1.781 df=4 | 0.1495 | ns | no | Two-tailed |
| 50 μM vs 100 μM | t=1.140 df=4 | 0.3177 | ns | no | Two-tailed |

**Quinine:**

|  | t, df | P value | P value summary | Are means signif. different? (P < 0.05) | One- or two-tailed P value? |
| --- | --- | --- | --- | --- | --- |
| DMSO vs 20 μM | t=6.104 df=4 | 0.0036 | ** | yes | Two-tailed |
| DMSO vs 50 μM | t=13.20 df=4 | 0.0002 | *** | yes | Two-tailed |
| DMSO vs 100 μM | t=7.606 df=4 | 0.0016 | ** | yes | Two-tailed |
| 20 μM vs 50 μM | t=0.3881 df=4 | 0.7177 | ns | no | Two-tailed |
| 20 μM vs 100 μM | t=3.393 df=4 | 0.0275 | * | yes | Two-tailed |
| 50 μM vs 100 μM | t=3.601 df=4 | 0.0227 | * | yes | Two-tailed |

**Clindamycin:**

|  | t, df | P value | P value summary | Are means signif. different? (P < 0.05) | One- or two-tailed P value? |
| --- | --- | --- | --- | --- | --- |
| DMSO vs 20 μM | t=1.917 df=4 | 0.1277 | ns | no | Two-tailed |
| DMSO vs 50 μM | t=6.909 df=4 | 0.0023 | ** | yes | Two-tailed |
| DMSO vs 100 μM | t=4.852 df=4 | 0.0083 | ** | yes | Two-tailed |
| 20 μM vs 50 μM | t=2.332 df=4 | 0.0801 | ns | no | Two-tailed |
| 20 μM vs 100 μM | t=0.7965 df=4 | 0.4703 | ns | no | Two-tailed |
| 50 μM vs 100 μM | t=2.240 df=4 | 0.0886 | ns | no | Two-tailed |

**Chloroquine:**

|  | t, df | P value | P value summary | Are means signif. different? (P < 0.05) | One- or two-tailed P value? |
| --- | --- | --- | --- | --- | --- |
| PBS vs 20 μM | t=0.4813 df=4 | 0.6555 | ns | no | Two-tailed |
| PBS vs 50 μM | t=0.7988 df=4 | 0.4692 | ns | no | Two-tailed |
| PBS vs 100 μM | t=2.061 df=4 | 0.1083 | ns | no | Two-tailed |
| 20 μM vs 50 μM | t=0.3286 df=4 | 0.7589 | ns | no | Two-tailed |
| 20 μM vs 100 μM | t=1.354 df=4 | 0.2473 | ns | no | Two-tailed |
| 50 μM vs 100 μM | t=0.8412 df=4 | 0.4476 | ns | no | Two-tailed |
